# Reply to: “Ipragliflozin improves the hepatic outcomes of patients with diabetes with NAFLD”

**DOI:** 10.1002/hep4.2006

**Published:** 2022-05-20

**Authors:** Laurens A. van Kleef, Robert J. de Knegt

**Affiliations:** ^1^ Department of Gastroenterology and Hepatology Erasmus MC University Medical Center Rotterdam the Netherlands

To the editor,

Despite the urgent need for effective nonalcoholic fatty liver disease (NAFLD) treatment and several novel agents being investigated, no drugs are yet approved. Hence, we read with great interest the article by Takahashi et al.: “Ipragliflozin Improves the Hepatic Outcomes of Patients With Diabetes with NAFLD.”^[^ ^1^ ^]^ The authors demonstrated in 46 patients with diabetes (subset endpoint 2) a beneficial effect of ipragliflozin (*n* = 21) on steatohepatitis and fibrosis.

The results of this randomized controlled trial are promising, as the disease burden of NAFLD increases rapidly. However, the reasons to power this study on a decrease of hemoglobin A1c are indistinct, as there is ample evidence available for this hypothesis and the authors focus on hepatic health.^[^ ^2^ ^]^ Suppose the hypothesis for power calculation was based on one of the most promising anti‐NAFLD drugs being investigated: The power was only 47% and 25% for detecting improvements in steatohepatitis and fibrosis, respectively.^[^ ^3^ ^]^ Studies with relatively low power need to be interpreted with caution, as they are prone to several issues, namely: (1) There is a lower chance of identifying genuinely true effects; (2) observed effects may not reflect true effects; and (3) estimates of identified true effects may be exaggerated.^[^ ^4^ ^]^

Additionally, randomization in small studies does not prevent all differences between subgroups, while statistical significance for these differences is typically not reached (because precision only increases with larger sample size).^[^ ^5^ ^]^ In this study, dyslipidemia appears to be more frequent in controls (*n* = 18; 72%) than in ipragliflozin users (*n* = 11; 52%). Assuming that lipid‐lowering treatment was only prescribed in patients with dyslipidemia, at best 22% (4 of 18) of controls and 36% (4 of 11) in the intervention group were treated according to guidelines. Because there is evidence for the hepatoprotective effect of statins in patients with NAFLD and dyslipidemia, these differences between the treatment and control group may have affected the observed effects.

Last, although we agree with the evident decrease of gamma‐glutamyltransferase (GGT) in patients using ipragliflozin, the levels at 72 weeks were not significantly different compared with the control group (50 U/L [30–64] vs. 52 U/L [36–72]). This may again be partially explained by “not‐significant differences” at baseline (68 U/L [38–114] vs. 54 U/L [36–87]), because among those with almost normal GGT, it is difficult to demonstrate a decrease.

Nonetheless, we are excited that ipragliflozin appears to have a range of hepatoprotective properties. Further studies should validate these findings in larger populations and address whether these hepatoprotective characteristics are generalizable to clinically relevant nondiabetes populations (e.g., prediabetes, metabolic syndrome).

## AUTHOR CONTRIBUTIONS

*Manuscript draft*: Laurens A. van Kleef. *Critical review of the manuscript and approval of final version*: Laurens A. van Kleef and Robert J. de Knegt. All authors approved the submission of the manuscript.

## FUNDING INFORMATION

Supported by the Foundation for Liver and Gastrointestinal Research, Rotterdam, the Netherlands
